# Whatman FTA cards versus plasma specimens for the quantitation of HIV-1 RNA using two real-time PCR assays

**DOI:** 10.1099/acmi.0.000138

**Published:** 2020-06-17

**Authors:** Abdourahamane Yacouba, Malika Congo, Gérard Komonsira Dioma, Hermann Somlare, David Coulidiaty, Kalifa Ouattara, Lassana Sangare

**Affiliations:** ^1^​ Laboratoire National de Référence du VIH/Sida et des Infections Sexuellement Transmissibles, Centre Hospitalier Universitaire Yalgado Ouédraogo, 03 B.P. 7022 Ouaga 03, Ouagadougou, Burkina Faso; ^2^​ Unité de Formation et des Recherches en Sciences de la Santé, Université Ouaga I Pr Joseph KI-ZERBO, 03 B.P. 7021 Ouaga 03, Ouagadougou, Burkina Faso

**Keywords:** Burkina Faso, dried blood spots, HIV-1, real-time PCR, viral load, Whatman FTA cards

## Abstract

**Background:**

Several studies have compared the use of dried blot spot (DBS) as an alternative to plasma specimens, mainly using Whatman 903 cards as filter paper. The aim of this study was to evaluate the use of Whatman FTA card (FTA card) specimens for HIV-1 viral load testing compared to plasma specimens using two real-time PCR assays manufactured by Roche and Abbott.

**Methodology:**

A cross-sectional study was conducted between April 2017 and September 2017 on HIV-1 patients admitted to Yalgado Ouédraogo Teaching Hospital. Paired FTA cards and plasma specimens were collected and analysed using the Abbott Real-Time HIV-1 assay (Abbott) and COBAS AmpliPrep/COBAS TaqMan v2.0 (Roche).

**Results:**

In total, 107 patients were included. No statistical differences (*P*>0.05) were observed between the mean viral loads obtained from the FTA cards and those of the plasma specimens using the Roche and Abbott assays. In total, 29 samples with Roche and 15 samples with Abbott assay showed discrepant results. At viral loads of ≤1000 copies ml^−1^, the sensitivity and specificity of the FTA cards were 78.6 and 100% with Roche, and 92.3 and 95.9% with Abbott, respectively. Both the Roche and Abbott assays showed good correlation and agreement between the FTA cards and plasma values.

**Conclusion:**

Our study demonstrates the feasibility of using FTA card filter paper for HIV-1 viral load testing. However, further studies will be required for the validation of the use of FTA card filter paper in HIV-1 treatment monitoring.

## Introduction

Viral load testing is the gold standard for HIV treatment monitoring. Periodic viral load tests are the most accurate way of determining whether antiretroviral therapy (ART) is suppressing viral replication [1–3]. With the use of ART expanding rapidly in resource-limited settings [3], testing viral load is a crucial step for scaling up the antiretroviral treatment. However, there are many barriers to viral load testing in resource-limited settings, including lack of basic essential equipment, as well as limitations in terms of the storage and transport of whole blood and plasma samples [4]. Due to the lability of viral RNA, whole blood in EDTA/K3 tubes cannot be stored for more than 6 h at 25 °C [5, 6]. Plasma storage and transportation require that plasma be transported within 24 h at 25 °C in EDTA/K3 tubes, or within 5 days at 4 °C for EDTA/K3 tubes, after centrifugation [7]. In low- and middle-income countries, these restrictive requirements for the transportation of whole blood and plasma mean that access to viral load testing is limited to those in close proximity to national or regional laboratories. Therefore, a simple method is needed to enable access to HIV-1 viral load testing for patients in rural areas.

Since June 2013, the World Health Organization (WHO) has recommended the use of dried blood spot (DBS) as an alternative to plasma for collection, transportation, HIV-1 viral load testing and genotyping drug resistance [8, 9]. DBS is an inexpensive and practical alternative to plasma since samples are easy to transport, without the need for cold chains or complex equipment. A further benefit of DBS is the reduction in blood sample volume [10, 11].

Numerous studies carried out in Burkina Faso and other countries have shown a strong correlation between DBS and plasma specimens for HIV-1 viral load testing [12–16]. However, in most of these studies, Whatman 903 card filter paper was the only filter paper used for HIV-1 load testing [17]. Diversifying the types of filter papers available for HIV-1 treatment monitoring could reduce the risk of shortages, decrease costs through price competition and increase the availability of filter paper. In Burkina Faso and other African countries, another type of paper, namely Whatman FTA card filter paper, is now routinely used for sample collection during malaria vigilance programmes and antimalarial drug trials [18].

Although Whatman FTA cards are also manufactured for blood sample collection, compared to Whatman 903 cards, they contain pre-dried chemicals that cause cell lysis in blood and protein denaturation and protect nucleic acids from nucleases, oxidative damage and UV damage during long-term storage (https://www.sigmaaldrich.com/catalog/product/sigma/whawb120205?lang=fr&region=FR).

The aim of this study was to evaluate Whatman FTA card (FTA card) specimens for HIV-1 viral load testing compared to plasma specimens using two types of assays: the COBAS AmpliPrep/COBAS TaqMan v2.0 HIV-1 test (Roche) and the Abbott RealTime HIV-1 assay (Abbott).

## Methodology

### Study site and design

A cross-sectional study was conducted between April 2017 and September 2017 at the National Reference Laboratory for HIV/AIDS and Sexually Transmitted Infections, located at Yalgado Ouédraogo Teaching Hospital (CHU-YO) in Ouagadougou, Burkina Faso. Socio-demographic, clinical and laboratory data were obtained from subjects using a structured questionnaire and laboratory analysis was performed on the blood samples.

### Study population

HIV-1 patients admitted for a follow-up visit at CHU-YO were enrolled as the study population. The inclusion criteria were as follows: (i) patients infected with HIV-1 who provided consent; (ii) antiretroviral-naive patients or patients under antiretroviral treatment. Patients who were positive for a fourth generation immunoassay screening test, confirmed by Western blotting, were defined being HIV-1-positive.

### Sample collection and processing

Whole blood was collected from the veins of the subjects and placed in two 4.7 ml EDTA/K3 tubes or a single 10 ml EDTA/K3 tube during their routine visits to CHU-YO. Before plasma separation, DBS was prepared by dispensing 50 and 100 µl of blood per spot (two spots per card) onto the FTA cards for the Roche and Abbott assays, respectively, and drying at room temperature (25±2 °C) for 18–24 h. The FTA cards were stored in zip-lock plastic bags with two silica gel desiccants at room temperature upon receipt. Plasma was obtained by centrifuging the whole blood, which was then aliquoted and stored at −70 °C until further testing for HIV-1 viral load. The FTA cards were then analysed not more than 14 days after being deposited. The FTA cards were processed for RNA elution according to the protocols provided by Roche and Abbott for HIV-1 RNA quantitation using the COBAS AmpliPrep/COBAS TaqMan v2.0 HIV-1 test and m2000rt, respectively.

For the Roche assay, a 12 mm punch of each DBS card was incubated with specimen extraction reagent and specimen pre-extraction reagent (SPEX) as a lysis buffer at 56 °C and 1000 r.p.m. for 10 min in a thermomixer. The samples were loaded into the Roche assay for automated HIV-1 RNA extraction for further real-time amplification detection.

For the Abbott assay, a spot (100 µl of blood) was obtained from each card for each specimen and placed in 15 ml Falcon tubes. Then, 1.4 ml of bulk m-lysis reagent provided with the Abbott sample preparation assay was added to each tube and incubated for 15 min with intermittent mixing at room temperature to perform lysis. The lysates (1 ml) were then transferred into tube S for manual HIV-1 RNA extraction, according to the manufacturer’s instructions. Real-time amplification and quantification were performed on the m2000rt assay system using the manufacturer’s protocol for 1 ml of DBS.

### Viral load quantification

The viral load was measured from the paired FTA cards and plasma specimens using the Roche and Abbott assays, according to the manufacturer’s protocols. The results of the viral load obtained from FTA card specimens were then compared to those of the plasma specimens.

### Statistical analysis

Statistical analyses were performed using RStudio (version 0.99.903). The sensitivity and specificity of the predictive positive value and predictive negative value were estimated to determine the performance of the FTA cards for the quantification of HIV-1 viral load at a viral load threshold of 1000 copies ml^−1^, a decision point for therapeutic efficacy. A Bland–Altman plot was generated to assess the limits of agreement and the mean bias [95 % confidence interval (CI)] in the viral load values obtained from the FTA cards and plasma specimens. Correlations between the viral loads obtained from the FTA cards and the plasma specimens were assessed using the Pearson statistical test. All HIV-1 viral load values were log_10_ copies ml^−1^ transformed prior to Bland–Altman and correlation analysis. The significance level was set at a *P* value (Fisher’s exact test) of 0.05 (*P*<0.05).

## Results

### Patient characteristics

In total, 107 patients were included in the study. The mean age of the patients was 42.0±13.4 years (ranging from 1 day to 77 years). The majority of the patients were female (sex ratio=0.39).

### Sample collection and bioanalysis

Whole blood was collected from all the patients. The paired FTA cards and plasma specimens collected were analysed using the Abbott and Roche assays for HIV-1 RNA viral load testing. Among the 107 paired FTA cards and plasma specimens tested, 8 FTA card specimens gave an invalid result with the Abbott assay and were excluded from further analysis. As a result, out of the initial 107 paired FTA cards and plasma samples collected, 99 samples were analysed.

### Comparison between FTA cards and plasma specimens in HIV-1 RNA quantitation

**Fig. 1.**
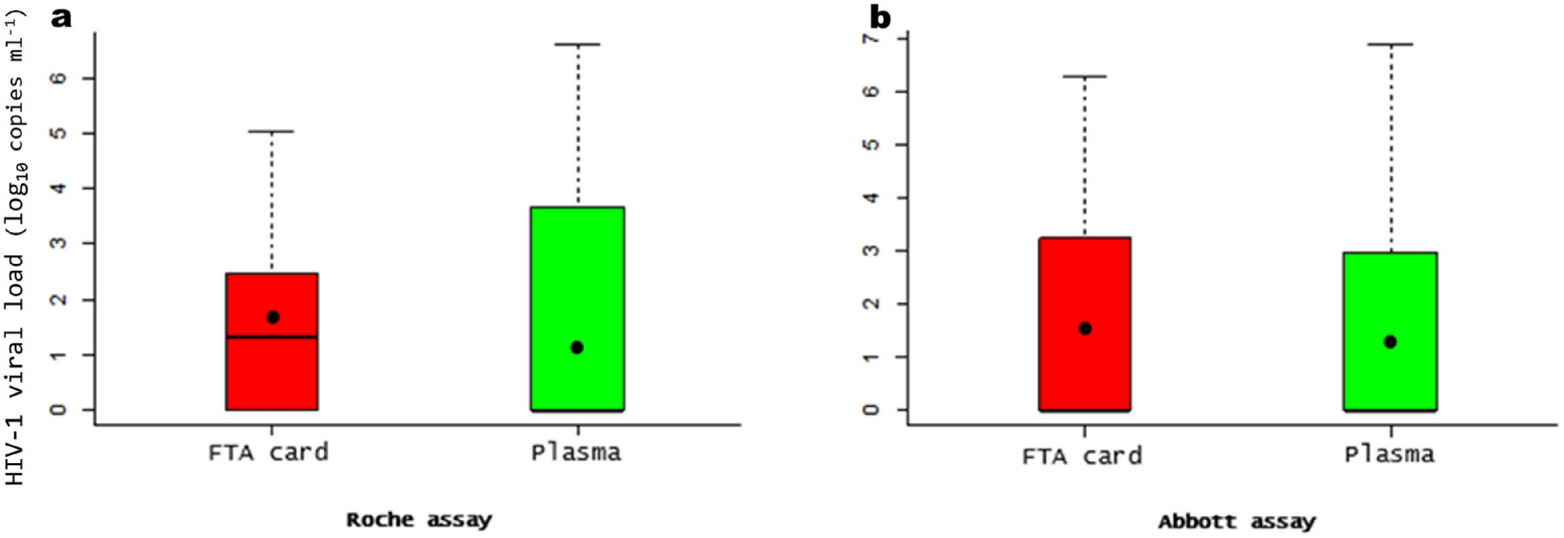
Comparison between Whatman FTA cards and plasma specimens in HIV-1 RNA. The boxplot to the left (a) using Roche assay; the boxplot to the right (b) using Abbott assay. The black points in the boxplot indicates the means values. Assay results are in log_10_ copies ml^−1^.

Using the Roche assay, no statistical differences (*P*=0.1704) were observed between the mean viral load obtained from the FTA cards (1.75 log_10_ copies ml^−1^) and plasma (1.37 log_10_ copies ml^−1^) specimens (Fig. 1a).

In total, 29 samples showed discrepant results. Eight (17.0%) of the samples tested were not detected on the FTA cards but showed moderately positive results (*n*=7; 14.9%) and high positive results (*n*=1; 2.1%) on the plasma specimens. Twenty-one (70.0%) samples tested moderately positive on the FTA card specimens but were not detected (*n*=16; 53.3%) or gave highly positive (*n*=5; 16.7%) results on the plasma specimens (Table 1).

**Table 1.** HIV-1 viral load using Whatman FTA cards and plasma specimens with the Roche assay. High, viral load ≥1000 copies ml^−1^; moderate, viral load <1000copies ml^−1^

FTA card specimens using Roche	Plasma specimens using Roche (%)			Total	*P* value
	Not detected	Moderate	High		
**Not detected**	39 (83.1)	7 (14.9)	1 (2.1)	47 (100.0)	1.5869e−17
**Moderate**	16 (53.3)	9 (30.0)	5 (16.7)	30 (100.0)	
**High**	0	0	22 (28.3)	22 (100.0)	

Using the Abbott assay, no statistical differences (*P*=0.72) were observed between the mean viral load obtained from the FTA cards (1.50 log_10_ copies ml^−1^) and the plasma specimens (1.38 log_10_ copies ml^−1^) (Fig. 1b).

In total, 15 samples showed discrepant results. Twelve (16.7%) samples were not detected on the FTA card specimens but were found to be moderately positive (*n*=7; 14.9%) and highly positive (*n*=2; 2.8%) on the plasma specimens. Three (11.1%) samples tested highly positive on the FTA card specimens but were not detected (*n*=1; 3.7%) or gave moderately positive (*n*=2; 7.4%) results on the plasma specimens (Table 2).

**Table 2.** HIV-1 viral load using Whatman FTA cards and plasma specimens with the Abbott assay. High, viral load ≥1000 copies ml^−1^; moderate, viral load <1000 copies ml^−1^

FTA card specimens using Abbott	Plasma specimens using Abbott (%)			Total	*P* value
	Not detected	Moderate	High		
**Not detected**	60 (83.3)	10 (13.9)	2 (2.8)	72 (100.0)	1.9542e−18
**High**	1 (3.7)	2 (7.4)	24 (88.9)	27 (100.0)	

### Performance of FTA cards for HIV-1 RNA quantitation

Using the Roche assay, the sensitivity and specificity of the FTA cards at a viral load of ≤1000 copies ml^−1^ were 78.6 and 100%, respectively (Table 3).

**Table 3.** Sensitivity, specificity, positive predictive value, and negative predictive value of Whatman FTA cards compared with paired plasma specimen for HIV-1 viral load testing at a 1000 copies ml^−1^ medical decision point

FTA cards (copies ml^−1^)		Plasma (copies ml^−1^)		Total	Se, Sp, PPV and NPV
		≤1000	≥1000		
**Roche**	**≤1000**	22	0	22	Se=78.6%; Sp=100.0 %, PPV=100.0%; NPV=92.2 %
	**≥1000**	6	71	77	
	**Total**	28	71	99	
					
**Abbott**	**≤1000**	24	3	27	Se=92.3%; Sp=95.9%; PPV=88.9%; NPV=97.2 %
	**≥1000**	2	70	72	
	**Total**	26	73	99	

Se, sensitivity; Sp, specificity; PPV, positive predictive value; NPV, negative predictive value.

Using the Abbott assay, the sensitivity and specificity of the FTA cards at a viral load of ≤1000 copies ml^−1^ were 92.3 and 95.9%, respectively (Table 3).

### Correlation and agreement between FTA cards and plasma specimens in HIV-1 RNA quantitation

**Fig. 2.**
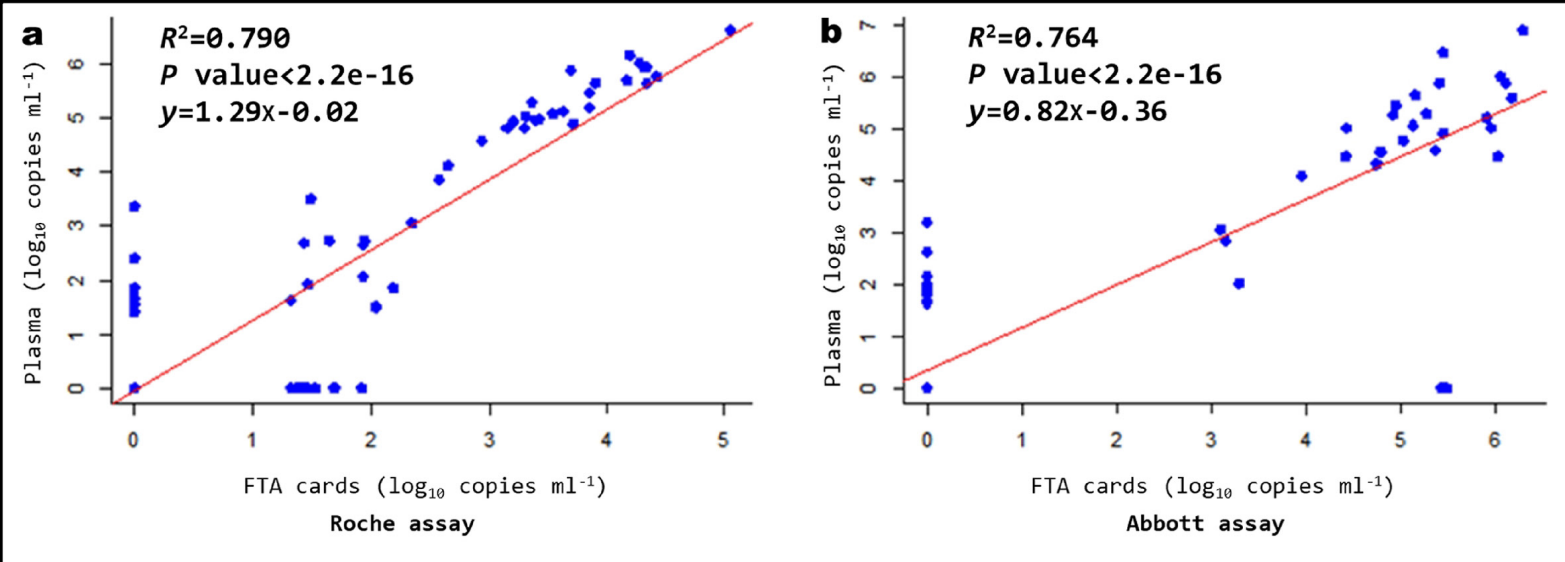
Correlation between FTA cards and plasma specimens in HIV-1 RNA quantitation. The red line indicates the best fit of the data to a linear regression. (a) Using the Roche assay. (b) Using the Abbott assay.

**Fig. 3.**
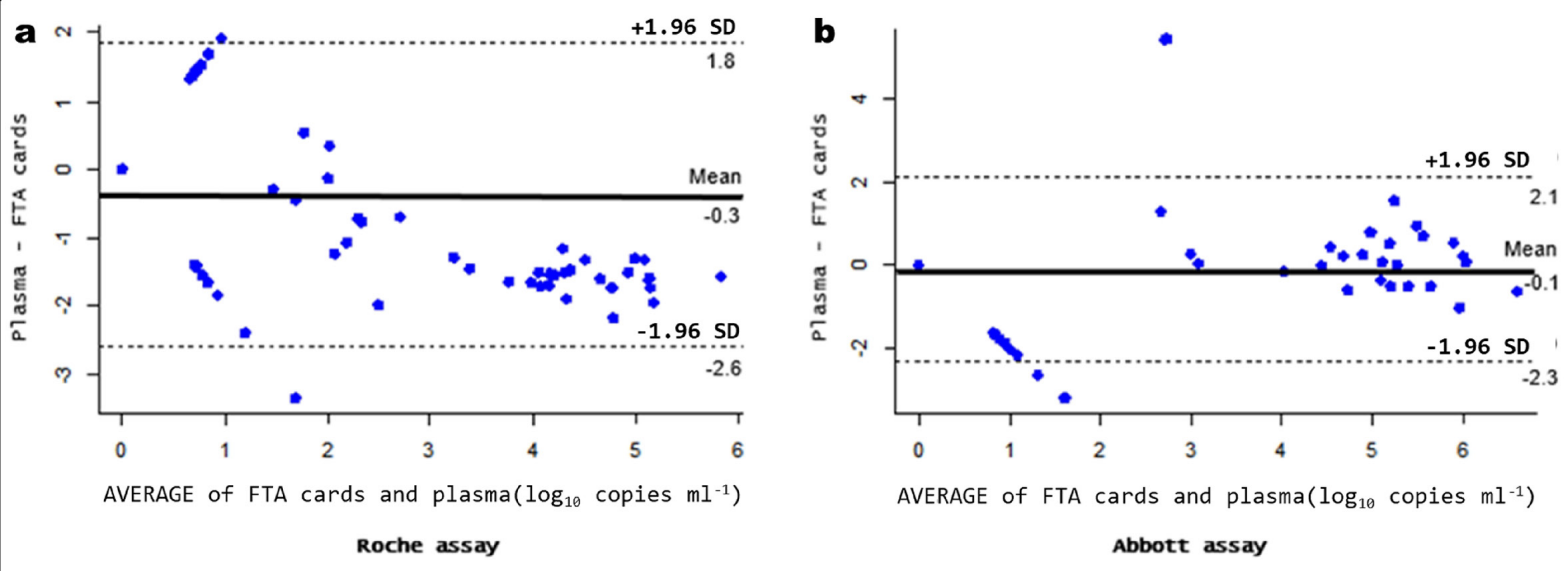
Bland–Altman analysis between FTA cards and plasma specimens in HIV-1 RNA quantitation. (a) Using the Roche assay. (b) Using the Abbott assay. The black line indicates the bias and the dotted black lines show 95% limits of agreement. Assay results are in log_10_ copies ml^−1^.

Using the Roche assay, we found a strong correlation (*R* ^2^=0.790; *P*<2.2e−16) between FTA cards and plasma specimen values (Fig. 2a). The Bland–Altman analysis showed a bias of −0.3 and 95% limits of agreement of −2.6 to 1.8 log_10_ copies ml^−1^; the total number of cases within the agreement limits was 97/99 (97.9 %) (Fig. 3a).

Using the Abbott assay, a strong correlation was obtained between the viral load values obtained from the FTA cards and the plasma specimens tested (*R* ^2^=0.764; *P*<2.2e−16) (Fig. 2b). The Bland–Altman analysis showed a bias of −0.1 and 95% limits of agreement of −2.3 to 2.1 log_10_ copies ml^−1^. The total number of cases within the agreement limits was 96/99 (96.9 %) (Fig. 3b).

## Discussion

Several studies have been conducted to compare the use of DBS as an alternative to plasma specimens, but they have mainly only used Whatman 903 as the filter paper [17]. In Burkina Faso, another type of paper (FTA cards) is also routinely used for sample collection during malaria vigilance programmes and antimalarial drug trials. In this study, the use of FTA cards was evaluated as an alternative method for plasma sample collection for HIV-1 RNA quantitation using commercial Roche and Abbott assays. To the best of our knowledge, this is the first study to evaluate and compare the use of FTA card filter paper (for DBS) for the collection of plasma specimens for viral load testing using both Roche and Abbott assays.

In our study, no statistical differences (*P*>0.05) were observed between the mean viral load obtained from the FTA cards and the plasma specimens using the two types of assays. These findings are similar to those of previous reports obtained using Whatman 903 cards [19–21]. However, in this study, 17.0% of the samples tested were not detected on FTA cards, but were positive for plasma specimens, with 2.1% of the samples being highly positive. These discrepant results are consistent with the findings reported by other studies using Whatman 903 cards [15, 22–24]. The reason for these discrepant results are well documented in the literature [15, 17, 22–24]. In a systematic review published in 2014, Smit *et al*. [17] indicated that the key reason for DBS not being as sensitive as plasma is due to the differences in sample volume between DBS and plasma. In the current study, the sample volume used on the FTA cards was 50 and 100 µl for the Roche and Abbott assays, respectively. Haematocrit has been suggested for the recalculation of the DBS viral load compared to the plasma viral load copies ml^−1^ by taking into account the differences between the plasma and DBS sample volumes [25, 26]. For this calculation, the haematocrit values need to be obtained to adjust the DBS viral load results by calculating the amount of plasma in the DBS samples. However, the present study did not use haematocrit adjustment. Further, according to the manufacturers’ protocols, haematocrit adjustment is not required for the calculation of viral load obtained from DBS when using the Roche and Abbott assays.

An overestimation of the HIV-1 RNA levels in the FTA card specimens with low-level viraemia (below 1000 copies ml^−1^) was observed in this study. This overestimation was also highlighted in the Bland–Altman analysis (mean difference of −0.3 and –0.1 log_10_ copies ml^−1^ in the Roche and Abbott assays, respectively). This observation is consistent with the findings reported by other research groups in terms of DBS [9, 17, 20, 22, 24]. The most advanced explanation of this repeated finding could be the contribution of intracellular HIV-1 DNA and RNA, which is present in the DBS but not in the plasma counterpart [27, 28]. Vidya *et al*. [19] suggested that the contribution of intracellular HIV-1 DNA and RNA could be more relevant to specimens with low or undetectable levels of viraemia than to specimens containing higher levels of extracellular HIV-1 RNA.

At the clinical threshold of 1000 copies ml^−1^, the sensitivity (Se) of the FTA cards was observed at 78.6% in the Roche assay, which was slightly lower than that observed in the Abbott assay (Se=92.3 %), according to the majority of the literature available using Whatman 903 cards [15, 22–25, 29]. In contrast, our results showed a higher value (Se=78.6 %) than those observed in Vietnam (Se=54.9 %) using Munktell TFN cards with the Roche assay [30]. Additionally, the contribution of HIV cell-associated DNA and RNA could account for the slightly lower sensibility in the Roche assay. Another possible explanation for the sensitivity observed in the Roche assay could be the elution protocol used in the present study. According to the manufacturer’s instructions, the time to incubation of DBS was 10 min in a thermomixer at 1000 r.p.m. and 56 °C.

Both the Roche and Abbott assays in this study showed good correlation and agreement between the FTA cards and plasma values, which was similar to other studies comparing DBS (using Whatman 903 cards) to plasma specimens using the Roche and Abbott assays [12, 14, 15, 21].

The present study had some inherent limitations. First, the sample size was restricted. Second, the FTA cards were not blotted via finger-pricked blood. Third, our study was a laboratory-based study, and therefore, the impact of the transportation on the FTA card samples was not evaluated. However, this study provides a preliminary insight into the design of a longitudinally designed study with a greater impact, incorporating additional factors, such as transport and storage under local conditions, to further evaluate FTA card specimens for HIV-1 viral load testing.

## Conclusion

In summary, this study demonstrates the feasibility of using FTA cards for HIV-1 viral load testing. FTA cards were found to be a sensitive and specific alternative to plasma testing for HIV-1 viral load testing using the Abbott assay. Both the Roche and Abbott assays showed a good correlation and agreement between the FTA cards and plasma values. These findings are relevant when considering how to improve access to viral load testing by diversifying the type of filter papers available in resource-limited settings.

In a future study, we will increase the testing population size and compare the use of Whatman FTA to Whatman 903 card specimens for viral load testing using both the Roche and Abbott assays.
